# The Gut Bacterial Resistome in the First Two Years of Life: Protocol for a Longitudinal Observational Birth Cohort Study

**DOI:** 10.2196/86058

**Published:** 2026-04-15

**Authors:** Justine Fri, Idris Njanje, Tjale Cloupas Mahopo, Lufuno Grace Mavhandu-Ramarumo, Pascal Obong Bessong, Microbiome and Antimicrobial Resistance Collaborators, Pascal Bessong

**Affiliations:** 1 SAMRC-UNIVEN Antimicrobial Resistance and Global Health Research Unit, Institute for Pathogen and Global Health Research, University of Venda, University Road, Private Bag X5050, Thohoyandou, 0950, South Africa, 27 15 962 8301; 2 Department of Nutrition, Faculty of Health Sciences, University of Venda, Thohoyandou, South Africa; 3 Department of Virology, Sefako Makgatho Health Sciences University, Pretoria, South Africa; 4 Center for Global Health Equity, University of Virginia, Charlottesville, VA, United States; 5 See Acknowledgments

**Keywords:** longitudinal birth cohort, antimicrobial resistance, bacterial gut resistome, ESKAPE pathogens, community AMR stewardship, Escherichia coli

## Abstract

**Background:**

Antimicrobial resistance (AMR) is a global health threat that increases the burden of infectious diseases and disproportionately affects communities of low socioeconomic status. Despite the call for community-level AMR data, prospective studies from rural sub-Saharan African communities to inform appropriate targeted interventions remain scarce. Given the role of enteric bacteria in AMR transmission dynamics, there is a need to understand the timing, risk factors, and ecological drivers of gut resistome acquisition and development during infancy.

**Objective:**

This study aimed to characterize the temporal dynamics of enteric bacterial resistomes during the first 2 years of life and to identify drivers of AMR acquisition and development in a community-based, prospective, observational birth cohort study in a rural South African community.

**Methods:**

The study aims to enroll 200 newborns and their mothers within 17 days post partum. Data on key exposures and variables include sociodemographics; perinatal and anthropometrics; feeding practices and dietary exposures; illness, medication, and vaccination history; breast milk metabolomic profiles; household socioeconomic status; maternal psychosocial and behavioral factors; hygiene and sanitation practices; and environmental exposures including hydro-meteorological variables, in-house livestock and pets, and drinking water quality. Biological samples include stools from monthly collections and diarrhea episodes for metagenomic analysis and breast milk for metabolomics. Planned analyses include assessing the infant microbiome and resistome structure (diversity, abundance, and composition) across time points and modeling associations between risk factors and AMR outcomes. Additionally, a cross-sectional community survey on knowledge, attitudes, and practices regarding antimicrobial use is conducted to inform knowledge translation through responsive dialogues, thereby developing ethnographically relevant packages for community-level AMR stewardship.

**Results:**

Participant identification and enrollment began in August 2023. By October 2025, 167 newborns had been enrolled, with 20 having completed the 24-month follow-up. The characteristics of the enrolled participants are presented in this protocol.

**Conclusions:**

This study will offer a unique opportunity to generate longitudinal resistome data from a rural sub-Saharan African setting. The study is expected to contribute knowledge on the microbiome and resistome structure dynamics and trajectories associated with key risk factors of acquisition and development. In addition, co-produced ethnographically tailored educational packages, informed by knowledge, attitudes, and practices and bacterial resistome data, will drive sustainable community-centered AMR awareness interventions.

## Introduction

Bacterial antimicrobial resistance (AMR) is a major threat to the treatment of infectious diseases and a significant contributor to morbidity and mortality worldwide. AMR disproportionately affects low- and middle-income countries. A systematic analysis revealed that in 2019 alone, bacterial AMR was responsible for close to 1.3 million deaths globally [1] and 250,000 deaths in the World Health Organization (WHO) African region alone [2]. The effects of AMR are reflected in poor health outcomes, absenteeism from work and school, an increase in health care expenditure for families and the state, and a decline in food security [3,4]. A concerted effort to mitigate the spread of antibiotic resistance is of high priority, and it is required to achieve certain sustainable development goals and global health imperatives. However, more often, efforts to tackle this challenge are concentrated in health care settings. The WHO working group on pathogen priority list of antibiotic resistance has emphasized the dearth of prospective data on the burden of antibiotic resistance from communities in low- and middle-income countries, to guide discovery and interventions [5]. Available datasets have limited power to contribute to national and regional understanding of AMR burden at the community level, and for policy development [6]. In addition, sustaining efforts to mitigate AMR through AMR stewardship requires investments in communities for the co-production and implementation of evidence [7,8].

The nonjudicious use of antibiotics in human and animal health, and the application of antibiotics in agriculture, drive the development of antibiotic resistance [9]. Poor infection control and prevention, poor hygiene and sanitation, and inadequate wastewater treatment and disposal contribute to the spread of AMR in the environment and its transmission to humans. Of interest is the relationship between changing meteorological conditions, such as higher temperatures, and the spread of pathogens, favoring human infection by hitherto mainly zoonotic microorganisms [10]. A multipronged approach is desirable to effectively mitigate AMR, given that it is driven by multiple factors impacting several dimensions of human life [11-13].

In a community-based cross-sectional study, we observed multiclass antibiotic-resistant *Escherichia coli* in children less than 4 months old with no prior exposure to antibiotics [14]. In the same community, it was later shown that enteroaggregative *E coli* was the most frequently exposed pathogen to antibiotics [15]. We have also identified bacterial taxa that could serve as indicators to monitor the repair of gut microbiota in malnourished children [16]. The human gut is a complex microbial ecosystem in which antibiotic resistance genes (ARGs) are embedded within microbial communities. A bacterial resistome may be shaped by the surrounding (“background”) microbial communities, as complex interactions between different bacterial species within these communities can create opportunities for horizontal gene transfer and contribute to resistome enrichment [17]. It is therefore plausible that the composition of the gut microbiota could influence the diversity and abundance of the gut bacterial resistome. To understand the risk factors and trends in enteric bacterial AMR, with the ability to measure the impact of interventions, *E coli* can be used innovatively as a model organism in apparently healthy populations. This is because *E coli* causes different diseases that require management with antibiotics, it is less fastidious, patterns of its resistome vary geographically, and it is a designated AMR priority organism.

For a better understanding of the landscape of enteric AMR to inform targeted interventions in communities of low socioeconomic status, the following questions need to be addressed: (1) At what age in a community of low socioeconomic status does the acquisition of enteric resistant *E coli* and other clinically relevant bacteria occur? (2) What is the composition of *E coli* resistomes and associated microbiota? Considering that several variables could contribute to the acquisition of enteric AMR, a plausible hypothesis is that the acquisition and carriage of resistant *E coli* and ESKAPE (*E coli* and *Enterococcus faecium*, *Staphylococcus aureus*, *Klebsiella pneumoniae*, *Acinetobacter baumannii, Pseudomonas aeruginosa,* and *Enterobacter spp*) bacteria begin early in life, and that colonization with resistant organisms is influenced by background microbiota and external exposures*.*

Reports have shown a strong association between hydrometeorological variables and enteric viral infections, and that earth observation data products, available at a global scale and at subdaily resolution, can be combined with longitudinal surveillance data to test hypotheses about the routes and drivers of transmission [18]. Also, it has been reported that the risk of infection with enteropathogens increases with an increase in soil moisture, and that humidity is associated with increases in bacterial infections [19]. If hydrometeorological variables impact the transmission of enteropathogens, they may also affect transmission dynamics and diversity of the enteric bacterial resistome. Therefore, do hydrometeorological indicators affect trends in the acquisition of community-level enteric resistomes? Using *E coli* as a model for the gut resistome, this question will be investigated through the following hypothesis: Seasonal changes in hydrometeorological indicators affect trends in the acquisition and composition of *E coli* resistome*.*

We have shown that rural low-socioeconomic communities can be prepared and organized to participate in intensive, prospective, observational studies [20]. Also, community-based projects can be leveraged to advance the sustainable development aspirations of communities [21]. Therefore, we hypothesize that engaged, involved, and mobilized communities are critical in the co-production of ethnographically relevant educational materials for antimicrobial resistance stewardship at the community level*.*

Considering the above hypotheses, the overarching aim of the study is to conduct a community-based, prospective birth cohort surveillance to understand the acquisition, composition, and evolution of enteric bacteria resistome. The specific objectives include the following:

1. To establish a prospective birth cohort of 200 mother and child pair participants.
2. To determine bacteria gut resistomes (total gut resistome and ESKAPE resistomes) in the first 2 years of life.
3. To determine risk factors of bacterial gut resistome acquisition, considering exposures and covariates.
4. Using *E coli* as a model to determine the association of background gut microbiota on gut resistome.
5. Using *E coli* as a proxy to study gut resistome to determine the impact of seasonal hydrometeorological indicators on *E coli* resistome.
6. To co-produce, with study communities, through knowledge translation and application, ethnographically relevant community-oriented AMR educational and awareness packages for community-level AMR stewardship.

The study, therefore, adopts a longitudinal observational cohort design with repeated follow-up from birth to 24 months of age, which allows direct observation of the timing, acquisition, and drivers of the infant gut resistome. Longitudinal mixed-effects and compositional analytical approaches are used to model temporal trajectories and assess associations between exposures and resistome outcomes. Shotgun metagenomic sequencing of repeated stool samples provides comprehensive outcome measures, including ARGs (abundance, diversity, and composition), as well as resistomes of sentinel organisms (*E coli* and ESKAPE pathogens) of public health importance. Detailed longitudinal data on maternal, infant, household, environmental, and hydrometeorological exposures are collected to align with the hypothesized drivers of resistome acquisition. Leveraging *E coli* as a model organism enables linkage of its resistome profiles with background microbiota signatures and the potential drivers. This approach provides a framework for generating locally relevant evidence on early-life acquisition of AMR with implications for AMR stewardship strategies at the community level.

## Methods

### Study Design

The primary design of the study is a prospective, observational birth cohort study to describe the acquisition, patterns, and burden of gut bacterial resistome in the first 24 months of life. This study protocol adheres to the STROBE (Strengthening the Reporting of Observational Studies in Epidemiology) guidelines for reporting observational studies [22], adapted to capture metagenomic-specific methodological details (STROBE-metagenomics) [23]. The STROBE checklist is provided in Checklist 1.

### Study Setting

The study is conducted in the Dzimauli community in the Limpopo Province, northeastern South Africa. The geography and climatic conditions of Dzimauli have been previously described [20,24]. Briefly, Dzimauli is rural and approximately 50 km from Thohoyandou, the main city of the Vhembe district, Limpopo Province. The population predominantly practices subsistence agriculture and petty trading. The area experiences heavy rainfall and flooding due to variations in climatic conditions [24].

### Sample Size

A sample size of 200 mother-child pairs was selected based on adequacy for the longitudinal study objectives. The cohort size is sufficient to estimate resistome carriage and burden, and to support mixed-effects and compositional analyses of resistome trajectories and associated exposures across repeated measurements at the community level. It also accommodates anticipated attrition (15%‐20%) over the 24-month period, while maintaining analytical robustness and feasibility. Although conducted in a small rural community, recruiting up to 200 participant pairs with extensive follow-up would enable population-level interpretation of findings or application of the study approach within a similar setting.

### Study Participants and Recruitment

Pregnant women in their third trimester are identified through household and outpatient antenatal clinic visits. Upon consent, they are screened and enrolled in the study at the birth of the child. Inclusion criteria for newborns include (1) participant should be available in the study community for at least 6 months, (2) birth weight ≥1500 g, (3) absence of immediate postnatal hospitalization or treatment for severe illness, and (4) mothers should be aged at least 16 years. Infants are excluded if (1) the child is from a multiple pregnancy, (2) the mother has another child enrolled in the AMR study, (3) the mother plans to relocate from the community within 6 months of enrollment, or (4) the mother is unable to consent or declines participation.

### Data Collection

Field workers were recruited from the study community and trained to screen and enroll participants and conduct surveillance activities. Data collection spans a 24-month period and includes structured surveillance questionnaires, biological samples, and clinical assessments to capture longitudinal information on maternal and child health, feeding practices, growth, socioeconomic status, hygiene, and environmental exposures [25]. A comprehensive summary of data collection domains, time points, and laboratory analyses is provided in Table 1. Briefly, at enrollment (≤17 d), maternal and child demographic, perinatal, and anthropometric data, as well as treatment, hospitalization, and breastfeeding practices, are collected. Child anthropometrics are subsequently collected monthly for 12 months and quarterly for another 12 months. The study adopted instruments developed and used in the same community by the MAL-ED (The Etiology, Risk Factors and Interactions of Enteric Infections and Malnutrition and the Consequences for Child Health and Development) study [25]. However, for the purpose of this study, household visits were conducted once a week for 24 months, during which time data on illness, antibiotic use, and other treatments received, feeding patterns, and vaccines received were captured by a 7-day recall questionnaire.

**Table 1.** Schedule of data collection measures, time points, participant groups, and outcomes in the 24-month longitudinal birth cohort study.

Measures	Time point	Group	Type of assay/analysis/outcome
Surveillance			
Recruitment, screening, and enrollment	≤17 days	Mother, child	Inclusion in a prospective study
Baseline demographic	≤17 days	Mother, child	Maternal and child demographics, perinatal and anthropometrics
Socioeconomic characteristics	6, 12, 18, and 24 months	Mother	Questionnaire on socioeconomic variables
Maternal assessment	≤17 days, 3, 6, 9, 12, 15, 18, 21, and 24 Months	Mother	Anthropometry, medication, and behavioral characteristics
Hygiene practices	1‐12, 15, 18, 21, and 24 months	Mother	Measures of food, water, and hygienic practices
Sanitation	6, 12, 18, and 24 months	—	Measures of sanitation
Hydrometeorological variables	Monthly from 1‐24months	—	Mean monthly precipitation volume, daily total surface runoff, soil moisture, surface pressure, wind speed, relative humidity, solar radiation, specific humidity, and average daily temperatures
Nutrition and health			
Food security	≤17 days, 6, 12, 18, and 24 months	Mother	Household food security variables
Infant Feeding	1‐24 months	Child	Report on infant feeding practices Exclusive breastfeeding Complementary Report on dietary diversity
Infant Food Diary	9‐12, 15, 18, and 24 months	Child	Record of infant food consumption in 24 h period, food sources, nutrient intake and adequacy
Child Growth Measures	1‐12-, 15, 18, 21, and 24 months	Child	Infant growth trajectory
Psychosocial	7, 15, and 24 months	Mother	Perceived stress, depression symptoms, and anxiety
Vaccine and treatment	1‐12-, 15, 18, 21, and 24 months	Child	Vaccination compliance and medication history
Laboratory assessment			
Stool	1-week, 3-, 6-, 12-, and 18-month	Child, Mother	Gut resistome
Breastmilk	1‐12-, 15, 18, 21, and 24 months	Mother	Metabolomics
Drinking water sample	Once during the study	—	Household drinking water quality and zoonotic *E coli* resistance
Saliva	Once between 1‐2 years	Child	Drug transport and metabolism
In-house livestock and pet fecal samples	Once during the study	—	Zoonotic *E coli* resistance

Surveillance data are collected with the mother or primary caregiver at various timepoints as detailed in Table 1. For infants aged 1‐8 months, a qualitative 24-hour dietary recall is adapted from the Demographic and Health Survey, which was developed for the same community as part of the MAL-ED study [25]. The qualitative dietary recall complements the once-weekly dietary reports by capturing a broader variety of foods consumed, including specific fruits and vegetables, grains, roots, and animal source foods. A quantitative 24-hour recall, used in the same community from the MAL-ED study [26] was also used from 9 to 24 months, to estimate energy and nutrient intake from nonbreast milk foods during the weaning process. In addition, to assess the household food access insecurity status. To assess food access insecurity, our survey included the nine-question household food insecurity access scale adapted in 2006 by the Food and Nutrition Technical Assistance project for use in low-resource settings [27] and used in the same population in the eight-countryside MAL-ED study by Psaki et al [28]. On a monthly basis, we assess water, sanitation, and hygiene practices of mothers or caregivers, incorporating the 5 key principles and the practical steps to achieve universal access to quality care WHO standards [29], as well as indicators related to antibiotic use and potential drivers of antibiotic resistance. At baseline, 7, 15, and 24 months, we also administer a Self-Reporting Questionnaire screening instrument that effectively measures depression in postpartum women, an instrument previously developed and used in the same population [30]. The Self-Reporting Questionnaire comprises 20 items that assess psychological disturbances related to depressive symptoms occurring within the previous 4 weeks. Questions are answered with a simple “yes” or “no,” and trained field assistants administer the instrument.

Mothers are trained to collect stool and breast milk samples as required. A stool sample is collected from the child and mother at enrollment, and then monthly, and whenever there is diarrhea, for 12 months. Thereafter, stool is collected quarterly, and whenever there is diarrhea, for a further 12 months. Breast milk is collected at enrollment and monthly, and for as long as the mother breastfeeds, for a total duration of 12 months.

Environmental samples, including household drinking water and fecal matter from in-household livestock and pets, are collected once during the study to assess zoonotic *E coli* resistome. Hydrometeorological exposure data such as precipitation, temperature, humidity, and soil moisture will be extracted from version 2.1 of the Global Land Data Assimilation System.

### DNA Extraction and Sequencing

The main goal is to generate metagenomic sequence data using next-generation sequencing, bioinformatic, and correlational analyses to understand infant microbial composition and resistance dynamics. Total genomic DNA (gDNA) is extracted from infant stool samples using a QIAamp Fast DNA stool min Kit (Qiagen) following the manufacturer’s protocol, with the inclusion of a bead-beating step, to the protocol. Extracted gDNA is quantified with a Qubit dsDNA High Sensitivity Assay, and library preparation is conducted using the Nextera XT DNA Library Prep Kit (Illumina). Sequencing will be performed on the Illumina NextSeq 2000 platform with paired-end reads (2×150 bp) using the P4 flow cell.

### Analysis of Enteric Bacterial Resistomes and Associated Risk Factors

The study will first characterize the total gut resistome and then focus on *E coli* resistome, where the organism will be used as a proxy organism for monitoring AMR in the gut microbiota due to its ubiquity, early colonization in the infant gut, and well-characterized genomic features. In addition, the ESKAPE pathogens’ resistome will be included to broaden our understanding of clinically relevant bacteria. By leveraging *E coli* and ESKAPE pathogens as sentinel organisms, this study aims to provide a focused yet robust characterization of the early-life enteric resistome, enabling deeper insights into AMR acquisition in low-resource community settings.

### Metagenomic Sequence Analysis

#### Overview

Bioinformatic analysis of metagenomic data from stool samples includes quality control and filtering, removal of host contaminants, taxonomic classification of reads, metagenome assembly and binning of assembled contigs, evaluation of bin completeness and contamination, taxonomic classification of bins, detection of AMR genes, and quantification of gene abundance through read mapping. Pipelines for sequence processing include BBduk (quality filtering) [29], BBmap (host decontamination and read mapping) [30], Kaiju (read-level taxonomic classification) [31], MetaSPAdes (assembly) [32], MetaBat (binning) [33], CheckM (bin quality assessment) [34], GTDB-Tk (bin classification) [35], and AMRfinder (ARG detection) [36]. ARGs will be classified by antibiotic class and resistance mechanism. The resulting metagenomic data presents an opportunity to study the total gut resistome, resistomes of *E coli* and ESKAPE pathogens, and other South African priority pathogens such as *Salmonella* and *Shigella.*

#### Association of Enteric Gut Resistome and Potential Risk Factors

Based on the major objectives, key analysis will be to (1) characterize the infant gut microbiome in terms of taxonomic composition, within and between sample diversity, and longitudinal trajectories across the first 24 months of life; (2) resistome profiles by assessing the abundance, diversity, and distribution of ARGs, including the identification of key resistance determinants; (3) investigate associations between resistome outcomes and host of metadata variables (sociodemographics; perinatal and anthropometrics; nutritional exposures, and feeding practices; illness, medication, and vaccination; breastmilk metabolome; household socioeconomic status; maternal psychosocial and behavioral factors; hygiene and sanitation practices; environmental exposures including hydro-meteorological variables, in-house livestock and pets, and drinking water quality); and (4) evaluate the temporal dynamics of the microbiome and resistome by leveraging longitudinal sampling to assess population-level trends over time. This will uncover temporal patterns and trajectories in gut microbial and resistome dynamics that may inform future interventions for AMR mitigation in early life.

The R software (R Foundation for Statistical Computing) is used for statistical analyses and graphical representations [37]. Descriptive statistics is used to summarize the mother-child pair characteristics of the cohort. The microbiome and resistome profiles are characterized using alpha (eg, Shannon index and Simpson index) and beta diversity (eg, Bray-Curtis dissimilarity) metrics, ordination (eg, principal component analysis using centered log-ratio-transformation data) to visualize compositional structure, and permutational multivariate analysis of variance testing for group-based differences, such as mode of delivery or nutrition. Core microbiome and resistome features are defined as taxa or ARGs present in ≥90 % of samples within a group. These are used to identify persistent and dominant features across time and metadata categories. Longitudinal changes in microbiome and resistome composition are modeled using linear mixed-effects models to account for repeated measures. Dirichlet-multinomial modeling is used to capture compositional shifts over time. For ARG abundance, exponential decay models are fitted to assess temporal trends, particularly in early life, where ARG load is expected to decline with age. Associations between microbiome or resistome features and covariates (eg, birth type, gender, dietary profiles, and meteorological data) are assessed using multivariable regression models. Spearman correlation is used for nonparametric associations. Where appropriate, machine learning models (eg, random forests) are used to identify predictive relationships between metadata and resistome features.

Differential abundance of taxa and ARGs is assessed using compositionally aware methods such as Analysis of Compositions of Microbiomes with Bias Correction. These models account for sampling bias and compositional constraints inherent in metagenomic data. Finally, core ARGs and taxa will be compared across groups to identify key features associated with metadata and time. Where appropriate, missing nonbiological covariate data will be addressed using complete-case analysis or multiple imputation, depending on missingness, and sensitivity analyses will be performed to assess the robustness of findings based on assumptions.

### Knowledge, Attitude, and Practice on the Use of Antimicrobials

To generate data that will contribute to the development of relevant educational packages, a community survey on Knowledge, Attitude, and Practice (KAP) on the use of antimicrobials is underway. A previous study in the Dzimauli area (in the Thulamela municipality in the Vhembe District of South Africa) from 2009 to 2012 reported an estimated population of 9000 [19]. Assuming an annual growth rate of 2.23% based on the population growth rate calculated from the Thulamela municipality population from 2011 to 2022 [38], the population of Dzimauli was projected to be 12,256 by 2023. The sample size for the community KAP survey was estimated as 466 targeted participants using the single-proportion formula with finite population correction (n=12,256), assuming *P*=0.5, d=0.05, and 95% CI and assuming 80% data completeness. A structured questionnaire is used to collect information on participant demographics, knowledge of antibiotics and antibiotic resistance, as well as attitudes and practices related to antibiotic use. The survey is conducted door-to-door and is administered in English or Tshivenda (the local language), depending on the participant’s preference. Inclusion criteria are those aged 16 years or older. The KAP component consists of 29 closed-ended questions (14 knowledge, 7 attitude, and 8 practice-related items). There is also a question to provide sources of information on antibiotics and 2 open-ended questions on general views. A 4-point Likert scale ranging from “Strongly disagree” to “strongly agree” was adopted to capture participant responses to closed-ended questions. Responses will be analyzed only for participants with complete data sets. The responses will be collapsed into a dichotomous set based on correct or incorrect responses. Total scores will be used to classify “high” (≥80%), “moderate” (60%‐79%), and “low” (<60%) KAPs on antibiotic use [39,40]. Frequency distribution for each KAP will be determined to understand the level of KAP in the community regarding antibiotics. Logistic regression analysis will be conducted to identify factors associated with KAP regarding antibiotic use, and odds ratios with 95% CIs will be reported. Statistical significance will be set at a *P* value <.05.

Open-ended responses from KAP surveys will be analyzed using thematic analysis. Responses will be transcribed where necessary, coded inductively, and grouped into thematic themes related to antibiotic knowledge, perceptions, practices, and contextual drivers of inappropriate antibiotic use. Qualitative findings will be used to contextualize and interpret quantitative KAP. Integration of qualitative and quantitative data will be conducted informally during interpretation, to support knowledge translation and the co-production of ethnographically relevant AMR stewardship interventions.

In addition to the community KAP survey, an end-assessment KAP survey is administered at 24 months, or when a participant exits the study. This latter survey aims at evaluating whether participation in a longitudinal AMR study has influenced maternal KAP related to antimicrobial use. Table 2 summarizes the different KAP assessments, the target population, and timing.

**Table 2.** Summary of the Knowledge, Attitude, and Practice (KAP) surveys, target groups, time points, and assessment focus in the Dzimauli birth cohort study.

Measures	Time point	Group	Type of analysis or outcome
Community KAP	Once off	Community	General Community KAP on antibiotic use
End assessment KAP	24 months	Mothers	Impact of participation in a prospective study on KAP

### Development of Ethnographically Appropriate Educational Packages for AMR Awareness Through Responsive Dialogues and Knowledge Translation

This activity draws from the KAP and enteric bacteria resistome datasets. During conversation events, participants will share their first-hand experiences on AMR and collectively brainstorm ideas and co-create solutions to tackle AMR challenges. Engagement with stakeholders, such as policy and decision makers, will take place during symposiums, which began during the conception of the project. Policy and decision makers are drawn from district, provincial, and national government health agencies charged with infection prevention and control.

### Research Data Integrity

We describe here how the study guarantees data quality, ensures reproducibility, and the ethics of doing research by tracking research integrity, misconduct, and misuse of research findings. The principal investigator reviews informed consent forms on the day they are signed, for correctness and completeness, and stores them securely. The research assistants are trained on the study protocol, data collection tools, and ethical conduct of research prior to commencement of fieldwork and through ongoing refreshers as needed. Case report forms are reviewed within 24 hours and signed by a supervisor. Corrected data on case report forms remains legible, signed, and dated. Repeated data capture errors trigger re-training for the worker or the group. Field and laboratory supervisors hold weekly meetings with staff to review data quality plans, challenges, and progress. Monthly, a supervisor does 5% of the monthly assessments for quality assurance and performs a dry-run training. Approved data are captured in REDCap (Research Electronic Data Capture; Vanderbilt University) system. Devices for anthropometric measurements are maintained and calibrated as recommended. Biorepositories are fitted with temperature monitoring devices and are monitored daily.

A protocol deviation form is completed for every out-of-schedule field assessment, laboratory test, or missing data. Case report forms are scanned and backed up electronically. In total, 85% of completeness and accuracy is the minimum targeted threshold for all data points. Data management is governed by policies and ethics on data creation, storage, sharing, and access. Training on ethics, integrity, and responsible conduct of research emphasizes data falsification, fabrication, plagiarism, and the reputation of the study community, investigators, institutions, and funders.

Potential tangible risks related to research misuse include the risk of misuse of (1) personal data and identifiers of study participants, and (2) analysis of DNA sequence data for reasons other than those of the current research. Soon after consent to participate is obtained, all data collection, tests, and assessments will include a participant identifier on the data collection tool. Samples also have sample identifiers. Through this, participants are delinked from samples, assessments, and datasets. In addition, access to data is password-protected. Study investigators, field, and laboratory workers are trained on the responsible conduct of research involving human participants.

The study generates next-generation sequencing data potentially containing human genes. It is worthwhile to note that it is difficult to deidentify a genome, because a genome by itself is in many ways an identifier. However, in this study, investigators avoid examining parts of the generated sequences that are not of interest to the study in order to avoid incidental findings, such as stumbling on variants that are not of interest to the study but could be of interest to a study participant. Incidental findings pose a dilemma. If this sort of event arises, the matter will be referred to the institutional research ethics committee for guidance on how to proceed. Generally, misuse of data is governed and managed according to legislation and guidelines regulating access to personal information and protection of human dignity.

### Ethical Considerations

The study protocol has approval from the Human and Clinical Trials Research Ethics Committee of the University of Venda, South Africa (FSEA/22/MBY/04/0302). Permission was granted by the Limpopo Provincial Department of Health, South Africa, to access local health care facilities in the study community for identification of potential participants (LP_2023-03-014). A Memorandum of Understanding regulates the relationship with the Dzimauli Tribal Authority. Several meetings were held with the community leadership to obtain community approval prior to individual consent. A community advisory committee was established to guide the values of respect, honesty, fairness, and justice vis-à-vis community participation, protection, and expectations from the study. Individually signed informed consent is obtained for all forms of participation in the study. To protect participants’ privacy and confidentiality, specimens and surveillance data are stripped of personal identifiers and coded. Ethical principles according to the Helsinki Declaration are adhered to [41]. The study participants did not receive compensation.

## Results

### Study Progress and Timeline

The study was funded in June 2022. Ethical approval was obtained in February 2023 from the Human and Clinical Trials Research Ethics Committee of the University of Venda, South Africa (FSEA/22/MBY/04/0302). Recruitment of mother-child dyads commenced in August 2023, with an expected end date in June 2026. By October 2025, 167 mother-child dyads had been enrolled, and baseline demographic data collection had been completed for the enrolled participants. Stool sample collection continues in accordance with the study follow-up schedule. Laboratory DNA extraction commenced in January 2024, while sequencing of the baseline samples for all participants began in August 2025, with initial data quality control and descriptive analyses expected by May 2026. Comprehensive integrative resistome-microbiome and qualitative analyses are planned following completion of data collection in December 2027. Dissemination of the principal study findings is anticipated through peer-reviewed publications and structured community feedback activities between June 2026 and December 2028.

### Characteristics of Currently Enrolled Participants

**Figure 1.**
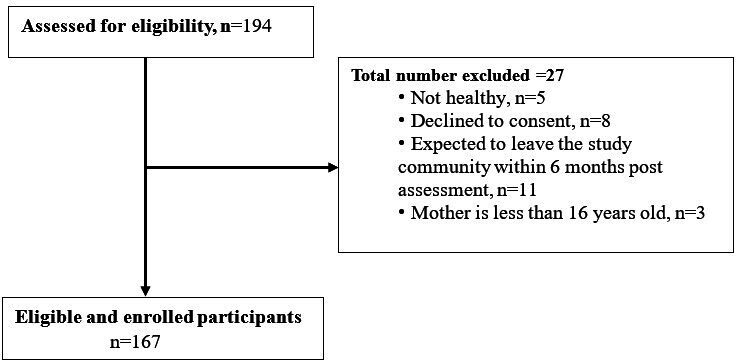
Flowchart of study cohort establishment: screening criteria and current enrollment.

Among the 167 newborns enrolled by October 2025, 20 participants have completed 24 months of follow-up. Baseline characteristics of these participants are summarized by total numbers with percentages and mean (SDs) to give an overview of participant characteristics. Proportions for each variable are based on the total number of participants with complete data on the assessment or question on the instrument. An attrition rate of 9.6% has been recorded. Figure 1 shows the enrolled strategy, while Tables3  4 show selected baseline descriptive characteristics of the 167 participant-dyads. The majority of the infants (129/167, 77.2%) were enrolled within 14 days after birth. There were no significant differences in sex (*χ*²_1_=0.15; *P*=.70) among enrolled babies, indicating an approximately equal representation of males and females. The mean weight of the babies at enrollment was 3.4 (SD 0.6) kg, compared to 3.1 (SD 0.5) kg recorded at birth. The mean length of babies at enrollment was 49.5 (SD 4.4) cm. None of the babies were hospitalized prior to enrollment, and 95.8% (160/167) received colostrum at birth. The length-for-age *z* scores indicated that 3.6% (6/165) were severely stunted, and weight-for-length *z* scores revealed a 5% (8/161) in the category of severe wasting. At enrollment, the mean age of the mothers was 27.7 (SD 7.1) years. Overall, 29.9 % (50/167) of the mothers had completed at least 12 years of schooling. Of those below 25 years, 30.6% (26/85) were attending at least high school. At enrollment, a baseline stool sample was collected from 96.4% (158/167) of the infant participants. For a robust statistical power calculation, a minimum threshold of 85% of complete baseline data collection for each of: sociodemographics, anthropometrics, treatment, hospitalization, and breastfeeding is being met.

**Table 3.** Baseline characteristics of enrolled participants.

Variables	Mean (SD)	Range (min-max)
Birthweight (kg)	3.1 (0.5)	1.9‐4.4
Weight at enrollment (kg)	3.4 (0.6)	2.1‐5
Length at birth (cm; n=160^a^)	49.0 (2)^b^	33‐57
Length at enrollment (cm)	49.5 (4.4)	33‐57
Head circumference at birth (cm; n=161^a^)	34.6 (1.60)	30‐38.1
Head circumference at enrollment (cm; n=166^a^)	35.3 (1.5)	30.6‐39
Child age (days)	9.6 (4.6)	1‐19^c^
Age of mother in years	27.7 (7.1)	16‐44
Number of live births of the mother	2.6 (1.6)	1‐8
Number of years of schooling of mother (n=164^a^)	11.1 (2.2)	2‐16

a N, when not equal to 167

b Median (IQR)

c There were 3 deviations from the target timeline of enrollment within 17 days of birth. (One infant was enrolled at day 19 after birth, and two infants at 18 days after birth).

**Table 4.** Distribution of enrolled infants and their mothers based on selected baseline variables.

Categories	Participants, n (%)
Gestational age at birth	
Term	160 (95.8)
Preterm	7 (4.2)
Infant sex	
Male	86 (51.5)
Female	81 (48.5)
Place of birth	
Health facility	163 (97.6)
Home	4 (2.4)
Birth weight group (kg)	
>2.5	144 (86.2)
1.9‐2.49	23 (13.8)
WLZ^a^ at enrollment (n=161^b^)	
−1≤ WLZ ≤1 (normal)	71 (44.1)
−2≤ WLZ <−1 (mild wasting)	22 (13.7)
−3≤ WLZ<−2 (moderate wasting)	9 (5.6)
WLZ < −3 (severe wasting)	8 (5)
1 <WLZ ≤2 (mild overweight)	41 (25.5)
2≤ WLZ ≤3 (overweight)	9 (5.6)
WLZ >3 (obese)	1 (0.6)
Length-for-age (LAZ)^c^ (n=165^b^)	
LAZ ≥ −1 (normal)	113 (68.5)
−2≤ LAZ<−1 (mild stunting)	32 (19.4)
−3≤ LAZ<−2 (moderate stunting)	14 (8.5)
LAZ < −3 (severe stunting)	6 (3.6)
Child fed first milk (colostrum)	
Yes	160 (95.8)
No	7 (4.2)
Prelacteal feeding	
Yes	67 (40.1)
No	100 (59.9)
Parity	
1 (low)	50 (29.9)
2‐3 (moderate)	73 (43.7)
>3 (high)	44 (26.3)
Mother’s marital status	
Never married	99 (59.3)
Married	66 (39.5)
Divorced	1 (0.6)
Widowed	1 (0.6)
Mother attended school	
Yes	164 (98.2)
No	3 (1.8)

a WLZ: weight-for-length *z* scores.

b N, when not equal to 167.

c LAZ: length-for-age *z* scores.

## Discussion

### Knowledge Gaps Being Addressed

This study protocol describes a prospective observational birth cohort designed to investigate the acquisition, composition, and transmission of ARGs during the first 24 months of life in a South African setting of low socioeconomic status. Early life exposures, particularly within the first 24 months of life, are critical for microbiome establishment and resistome development and shape long-term immunity, metabolism, and resistance profiles [31,32]. By integrating longitudinal metagenomic surveillance with external exposures, including hydrometeorological and contextual data, the study aims to address key gaps in understanding the temporal dynamics of microbiome-resistome and AMR emergence and dissemination outside health care settings. In parallel, the study incorporates KAP assessments on antibiotic use [33] and the co-production of intervention materials, with the goal of translating biological findings into contextually relevant AMR mitigation strategies [34].

### Potential Study Findings

Accordingly, a multidimensional longitudinal birth cohort in Dzimauli, a rural South African setting, as a model for community-level AMR stewardship is being established. The cohort is designed to integrate biological, social, and contextual data to provide comprehensive interventional data for community-level AMR. Integrative data generated from this cohort is expected to provide several important contributions to AMR evidence-based interventions. First, the study is expected to generate critical prospective datasets describing the enteric resistome in a low-socioeconomic community, addressing a major gap in community-level AMR surveillance. Longitudinal metagenomic enteric profiles will reveal critical data, including acquisition, composition, persistence, and evolution of ARGs; community-level intervention hotspots; emerging community-level critical pathogens of clinical relevance and potential transmission pathways, including vertical and horizontal transmissions within households and the broader environment. We capture a wide range of exposures, contextual factors, and potential confounders to clearly identify drivers of AMR. Integrative metagenomic and exposure data will provide intervention insights. This approach ensures that intervention strategies are grounded in local realities and informed by community perspectives. Finally, the study will ensure enhancement of the development of leadership in integrative AMR epidemiology in low-income communities for local and global relevance.

Current evidence on AMR in low-resource settings is dominated by cross-sectional studies, which provide only snapshots of resistance patterns at single time points [35,36]. Although such studies are valuable in terms of estimating prevalence, they are limited in their ability to capture proper AMR baseline and temporal dynamics data, including when ARGs are first acquired, which bacteria greatly contribute the most ARGs, how they persist or change over time, and how their transmission pathways evolve. The emergence of AMR itself is complex, shaped by the interaction of biological, environmental, behavioral, and social factors [37,38]. However, a plethora of studies focus on a limited subset of potential drivers, such as antibiotic use or selected environmental variables, without accounting for broader contextual influences [39,40,42]. This narrow focus may bias interpretations of AMR drivers and underestimates the contribution of diverse structural and social determinants. Furthermore, while many AMR studies generate detailed microbiological data, limited studies explicitly link these findings to community knowledge, behaviors, lived experiences, and the production of setting or regional-specific interventions [34,43,44]. These limitations are particularly pronounced in sub-Saharan Africa, where longitudinal AMR data from community settings, especially among infants and young children, remain scarce [45]. As a result, the establishment of an interdisciplinary birth study cohort is key to addressing these limitations and generating the longitudinal, contextualized data necessary for effective AMR surveillance and stewardship.

### Community-Based Challenges

It is worthwhile to indicate challenges faced so far in the cohort establishment, biospecimen and surveillance data collection, and the strategies being applied to mitigate the challenges. First, due to socioeconomic factors, there is a fair number of relocations of community residents out of the study area. Therefore, effort is required to identify potential eligible participants who aim to stay long enough. This approach contributes to lower attrition and meaningful contributions to the datasets envisaged. Second, the topology of the study community makes the mobility of the field workers to access households difficult. To address this, efforts were made to increase the number of field workers such that the number of households that each field worker is responsible for is reduced. The added resources to address the challenge of mobility pay off by enhancing timely data collection, completeness, and accuracy. Third, the display of fatigue in providing samples prospectively. This behavior was diagnosed as an inadequate understanding of why a specimen, for example, stool, is collected repeatedly. This is addressed through conversations with relevant participants for the purpose of repeated collection and through continuous consenting.

### Strengths and Limitations

The cohort study has major strengths, including its (1) prospective design and community-based nature, which address a critical evidence gap in community engagement and co-production of scientific evidence in the mitigation of AMR, (2) its broad longitudinal exposure data, including the integration of hydrometeorological and nutritional variables, would enable a holistic assessment of potential drivers of resistome acquisition beyond antibiotic exposure alone, and (3) the use of shotgun metagenomic sequencing to determine the resistome makes it a strong methodological approach to obtain large datasets necessary for the discovery of novel niches and relevant priority organisms driving AMR. Overall, it brings to the fore the role of enteric resistome in child health and livelihoods for global health imperatives.

Despite these strengths, the cohort study should be considered with certain potential limitations. These include the introduction of recall bias, increased risk of missing or delayed data due to the prospective nature and the intensity of data collection; factors such as undocumented exposures, such as over-the-counter antibiotic use, may be missed; the management of attrition rate in some communities, for example, pastoral communities, may be difficult. Furthermore, although metagenomic data provides a high level of granularity, it may not be feasible in resource-constrained research environments.

### Conclusions

Large prospective datasets on AMR are necessary for decision-making and intervention in sub-Saharan African settings, particularly in low-socioeconomic settings. Therefore, a comprehensive assessment of the multiple and interacting determinants that drive AMR dynamics remains an essential aspect in AMR stewardship. This protocol is a proposal for AMR research at the community level to identify drivers important for AMR mitigation involving local communities in data and evidence generation.

## Supplementary material

10.2196/86058 Checklist 1 STROBE checklist.

10.2196/86058 Peer Review Report 1 Peer-review report from the South African Medical Research Council (SAMRC).

## Abbreviations

AMR: antimicrobial resistance
ARG: antibiotic resistance gene
ESKAPE: *Enterococcus faecium, Staphylococcus aureus, Klebsiella pneumoniae, Acinetobacter baumannii, Pseudomonas aeruginosa, and Enterobacter spp.*
KAP: Knowledge, Attitude, and Practice
MAL-ED: The Etiology, Risk Factors, and Interactions of Enteric Infections and Malnutrition and the Consequences for Child Health and Development
REDCap: Research Electronic Data Capture
STROBE: Strengthening the Reporting of Observational Studies in Epidemiology
WHO: World Health Organization
