# Supplementary material for: The Gut Bacterial Resistome in the First Two Years of Life: Protocol for a Longitudinal Observational Birth Cohort Study
Source: JMIR Res Protoc. 2026 Apr 15;15:e86058. doi: 10.2196/86058 (PMC13082449; doi:10.2196/86058)
Supplement: Peer Review Report 1 [file resprot-v15-e86058-s002.pdf]

Dear Editor-in-Chief

JMIR Research Protocols

I wish to indicate that this study is partially funded through a grant application submitted to the South African Medical Research Council (SAMRC). However, no formal reviewer reports are available. Below is proof of support from the SAMRC.

Sincerely

Pascal O. Bessong, PhD, mASSAf

Director, SAMRC-UNIVEN Antimicrobial Resistance & Global Health Research Unit

University of Venda, Private Bag X5050, Thohoyandou 0950, South Africa

Email: pascal.bessong@univen.ac.za

Tel: +27 15 962 8301

**OFFICE OF THE VICE PRESIDENT:**
VP-ERIP

**Prof Pascal Bessong**
Department of HIV/AIDS & Global Health Research Programme
Faculty of Science
University of Venda
Limpopo

24 February 2022

bessong@univen.ac.za

Dear Professor Bessong

**Outcome of RFA (SAMRC-RFA-EMU-06-2021) on establishment of new Extramural Units (EMUs) – SAMRC/UNIVEN Antimicrobial Resistance and Global Health Research Unit.**

Thank you for submitting an application for the establishment of a new SAMRC Unit. I am pleased to inform you that your application was **successful**.

The recommendations of the peer reviewers were discussed at the SAMRC Executive Management Meeting (EMC) held on 27 January 2022 and approved by the SAMRC Board on the 24th February 2022. It was unanimously agreed to award the SAMRC/UNIVEN Antimicrobial Resistance and Global Health Research Unit - effective from 1 April 2022. This was based on your outstanding scientific stature as a researcher who will continue to add value to the SAMRC strategic direction.

As you are aware, the funding for the SAMRC EMUs represents a secure, discretionary, financial incentive which is approved in 5-year cycles for a potential lifespan of 15-years depending on the EMU performance and should facilitate efforts to leverage external research funding. It is expected that institutional co-funding and the sum of external grants should exceed several-fold the amount invested by the SAMRC in the Unit.

The SAMRC congratulates you on your new and well-deserved role as Director of the Unit. We trust you will continue to provide excellent leadership for the new Unit to achieve exceptional research performance and output.

OFFICE OF THE
VICE PRESIDENT:
VP-ERIP

Below is the feedback from the Reviewers to help you and your research team in strengthening and improving the potential impact of your proposed research plan.

We wish you and your team well in your research endeavours.

**Sincerely,**

**Prof Liesl Zühlke**
**Vice President: Extramural Research & Internal Portfolio**

cc: Professor Glenda Gray, President and CEO, South African Medical Research Council
Dr Refiloe Eunice Zwane, Senior Programme Manager: Vice President's Office, SAMRC

THE SOUTH AFRICAN MEDICAL RESEARCH COUNCIL
Francie van Zijl Drive, Parowvallei, Cape Town, PO Box 19070, South Africa
Tel: +27 12 339 8640 | Email: liesl.zuhlke@mrc.ac.za

## Feedback from the Peer Review Process

<u>Excerpts from different Reviewers</u>

*"This is a proposal to develop an SAMRC EUA focused on AMR, broadly defined. The EUA will be led by an energetic, established investigator focused on two broad areas of (his) longstanding interest: HIV and infant microbiome. The investigator describes a team that seems capable of forming and following required cohorts and studying specimens collected. The unit will generate observational data that they propose can/will be used to inform and influence policy makers, and to inspire study communities to better health outcomes."*

*"A strong proposal by a strong team. Overall, I rate this proposal highly and consider the team to be demonstrably competent in delivering to the objectives outlined. I have some questions on how the proposal could be perhaps strengthened. The study for major aim 3: is the population only new-borns or their mothers as well? It would be important to include their mothers as well as mode of delivery as a confounder for E-coli in the microbiota. I am sure the applicants will consider these factors."*

*"Prof Bessong has more than 15 years experience working on HIV drug resistance and more recently on E. coli microbiome and resistome in sick and healthy children. He has focused his research in an area that is of huge public health significance in SA and the region. He proposes to use new technologies to provide data on surveillance for HIV drug resistance as well enabling public health policy makers access data on microbiome changes that are biomarkers for child health and wellness. He has proposed a robust program that encompasses both research and training, thus enabling an environment to enhance human capital that will provide continuity for the proposed activities. I highly recommend funding to this initiative to create the new unit of Antimicrobial resistance and global health."*
